# Draft genome sequence of *Chryseobacterium endophyticum* strain Cas268 isolated from rhizosphere soil in China

**DOI:** 10.1128/mra.00621-26

**Published:** 2026-07-16

**Authors:** Baoxiang Pan, Yueqi Xu, Xiaobing He, Jinchang Liang, Xiaoqiang Wang, Tianbo Liu, Tiansheng Zhu

**Affiliations:** 1 College of Agriculture, Tarim University 12483, Alar, China; 2 State Tobacco Monopoly Administration (China National Tobacco Corporation), Key Laboratory of Tobacco Pest Monitoring Controlling & Integrated Management, Tobacco Research Institute of Chinese Academy of Agricultural Sciences, Qingdao, China; 3 Pingdingshan Tobacco Company of Henan Tobacco Monopoly Bureau, Pingdingshan, Henan, China; 4 Hunan Tobacco Science Research Institute, Changsha, China; DOE Joint Genome Institute, Berkeley, California, USA

**Keywords:** draft genome, *Chryseobacterium endophyticum*, rhizosphere soil

## Abstract

The bacterial strain *Chryseobacterium endophyticum* Cas268 was isolated from tobacco rhizosphere soil in China. In this study, we report the draft genome sequence of this strain. The genome measures 4,299,272 bp in length and has a G + C content of 39.56%.

## ANNOUNCEMENT

Bacteria of the species *Chryseobacterium endophyticum* are gram-negative, aerobic, and non-motile, and they belong to the family Weeksellaceae (1). These organisms are frequently encountered in a variety of environments, such as soil, water, and plant-associated habitats. Certain members within this genus are known to function as plant growth-promoting rhizobacteria (2). To support genomic investigations into a beneficial *Chryseobacterium* strain, we performed the genome sequencing of *C. endophyticum* Cas268 (2).

The bacterial strain designated Cas268 originated from rhizosphere soil collected in a tobacco field located in Hubei Province, China, on August 15, 2023. Rhizosphere soil was defined as the soil tightly adhering to the roots after gentle shaking to remove loose bulk soil, then scraped from the root surface. Isolation of the strain was carried out through serial dilution and subsequent plating onto nutrient agar (NA) (3), with incubation at 28°C for 72 h. A single yellow-pigmented, smooth, circular, convex colony with entire margins was selected and purified by repeated streaking onto fresh NA plates (4). Initial taxonomic identification was achieved via PCR amplification and Sanger sequencing of the 16S rRNA gene, employing the universal primers 27F and 1492R (5). The obtained sequence (accession number PZ158634) was analyzed using EzBioCloud (https://www.ezbiocloud.net/). Based on 16S rRNA gene sequence analysis, isolate Cas268 showed 99.10% similarity to *C. endophyticum* CC-YTH209^T^ (KU358716), 97.57% to *C. endophyticum* CC-CZW010^T^ (KM206767), and 97.29% to *C. rigui* CJ16^T^ (JQ071497), confirming that strain Cas268 belongs to *C. endophyticum*.

Strain Cas268 was cultured in nutrient broth at 28°C for 24 h (6). Genomic DNA was subsequently isolated using a phenol-chloroform extraction method (7). The purity and concentration of the extracted DNA were assessed via agarose gel electrophoresis and Qubit fluorometry, respectively (8). A sequencing library was constructed using the NEBNext Ultra DNA Library Prep Kit (New England Biolabs, USA) and sequenced on an Illumina NovaSeq X Plus instrument (Illumina, San Diego, CA), yielding an average insert size of 270 bp (9). The run produced 4,569,005 paired-end reads (150 bp each) (1.58 Gb), corresponding to an estimated 369-fold coverage of the genome. Quality filtering of the raw Illumina reads was performed with fastp, removing reads in which more than 40% of bases fell below a quality score of 15 (10). The remaining high-quality reads were assembled *de novo* using SPAdes version 3.7, and any contigs shorter than 1,000 bp were discarded from the final assembly (11). Unless otherwise specified, all software was executed with default parameters.

The assembled genome of strain Cas268 comprises 35 contigs with a total length of 4,299,272 bp, with an N50 of 387,357, and a G + C content of 39.56% (Table 1). Genome annotation was performed using the NCBI Prokaryotic Genome Annotation Pipeline, which annotated 3,866 protein-coding genes (12). In addition, tRNA genes were predicted with tRNAscan-SE v1.3.1 (13), and rRNA operons were identified using RNAmmer v1.2 (14). The genome completeness and contamination were estimated to be 100% and 1.11%, respectively, using CheckM2 v1.1.0 (15). The genome was predicted to contain 54 tRNAs and 3 rRNAs.

**TABLE 1:** Summary of genome information

Parameters	Value
Contigs	35
Coverage (×)	369
Genome size	4,299,272 bp
G + C content	39.56%
Coding sequences	3,897
Protein-coding genes	3,866
tRNA genes	54
5S rRNA genes	1
16S rRNA genes	1
23S rRNA genes	1
Completeness	100%
Contamination	1.11%

## Data Availability

This Whole Genome Shotgun project has been deposited at GenBank under accession number JBWGXE000000000. The version described in this paper is the first version, JBWGXE000000000.1. The raw sequencing reads have been deposited in the NCBI Sequence Read Archive (SRA) under accession number SRR37570553.
